# Heterologous production of caffeic acid in microbial hosts: current status and perspectives

**DOI:** 10.3389/fmicb.2025.1570406

**Published:** 2025-04-29

**Authors:** Yuanzi Li, Jiaxin Li, Miao Zhang, Yonghong Liao, Fenghuan Wang, Mingqiang Qiao

**Affiliations:** ^1^School of Light Industry Science and Engineering, Beijing Technology and Business University (BTBU), Beijing, China; ^2^Beijing Advanced Innovation Center for Food Nutrition and Human Health, Beijing Technology and Business University (BTBU), Beijing, China; ^3^The Key Laboratory of Molecular Microbiology and Technology, Ministry of Education, College of Life Sciences, Nankai University, Tianjin, China; ^4^College of Life Sciences, Shanxi University, Taiyuan, China

**Keywords:** caffeic acid, microbial production, pathway engineering, metabolic engineering, synthetic biology

## Abstract

Caffeic acid, a plant-derived phenolic compound, has attracted much attention in the fields of medicines and cosmetics due to its remarkable physiological activities including antioxidant, anti-inflammation, antibacteria, antivirus and hemostasis. However, traditional plant extraction and chemical synthesis methods exist some problems such as high production costs, low extraction efficiency and environmental pollution. In recent years, the construction of microbial cell factories for the biosynthesis of caffeic acid has attracted much attention due to its potential to offer an efficient and environmentally-friendly alternative for caffeic acid production. This review introduces the caffeic acid biosynthesis pathway first, after which the characteristics of microbial hosts for caffeic acid production are analyzed. Then, the main strategies for caffeic acid production in microbial hosts, including selection and optimization of heterologous enzymes, enhancement of the metabolic flux to caffeic acid, supply and recycling of cofactor, and optimization of the production process, are summarized and discussed. Finally, the future prospects and perspectives of microbial caffeic acid production are discussed. Recent breakthroughs have achieved caffeic acid titers of up to 6.17 g/L, demonstrating the potential of microbial biosynthesis. Future research can focus on the enhancement of metabolic flux to caffeic acid biosynthesis pathway, the development of robust microbial hosts with improved tolerance to caffeic acid and its precursors, and the establishment of cost-effective industrial production processes.

## 1 Introduction

Caffeic acid (3,4-dihydroxycinnamic acid) is a natural phenolic compound widely existed in plants such as *Crataegus pinnatifida*, *Thymian*, *Solidago virgaurea*, and *Eucommia ulmoides* (Touaibia et al., 2011; Yang et al., 2013; Espindola et al., 2019). It has attracted much attention in the fields of medicines and cosmetics due to its demonstrated pharmacological actions including antioxidant, anti-inflammation, antibacteria, antivirus, hemostasis, analgesis, prevention of cardiovascular disease, protection of brain tissue damage, and promotion of wound healing (Touaibia et al., 2011; Khan et al., 2016; Alson et al., 2018; Teng et al., 2020; Pavlikova, 2022). Moreover, its derivatives like caffeic acid phenethyl ester and chlorogenic acid also have important pharmacological activities (Wang et al., 2017; Xiao et al., 2022).

According to statistics and forecasts by QYResearch, the global market sales of caffeic acid reached 0.13 billion USD in the year 2024 and is expected to reach 0.16 billion USD by the year 2030, with a compound annual growth rate of 3.8% from 2024 to 2030. Caffeic acid is commonly extracted from plant sources, but its low accumulation in plants makes the separation process both difficult and expensive to some extent (Ye et al., 2010). Additionally, extraction methods involve high-temperature treatments, the use of petroleum ether, and solvent extraction, which are energy-intensive and environmentally unfriendly (Zhang et al., 2014; Rodrigues et al., 2015; Han et al., 2024; Qian et al., 2024). Caffeic acid can also be produced by chemical synthesis, but the application of this technique is greatly limited by the requirement for toxic solvents, the complex production process and the production of byproducts (Zhang et al., 2014). In recent years, the development of synthetic biology techniques and metabolic engineering make it possible to produce plant-specific secondary metabolites in heterologous microbial hosts. The use of microbial hosts has made significant contributions to the biosynthesis of industrial and pharmaceutical compounds due to its advantages of low production cost, high product purity, high efficiency, environmental friendliness, and the simplicity of the genetic manipulation process (Marienhagen and Bott, 2013; Davy et al., 2017; Cravens et al., 2019). Heterologous production of caffeic acid in microbial hosts provides a feasible way to produce caffeic acid and has attracted much attention of researchers. The first report on microbial production of caffeic acid was achieved in *Streptomyces fradiae* XKS (Berner et al., 2006). Subsequently, more and more studies have been carried out, and the concentration of caffeic acid has reached 6.17 g/L through the introduction of its heterologous biosynthetic pathway and the metabolic engineering of the microbial host (Sakae et al., 2023).

This review summarizes the recent advances in the heterologous production of caffeic acid in microbial hosts, discusses the tools and strategies employed at the gene, protein, and pathway levels, as well as the optimization of the fermentation process to achieve efficient biosynthesis of caffeic acid. Furthermore, the future opportunities and challenges in the production of caffeic acid is also discussed.

## 2 Caffeic acid biosynthesis pathway

**Figure 1:**
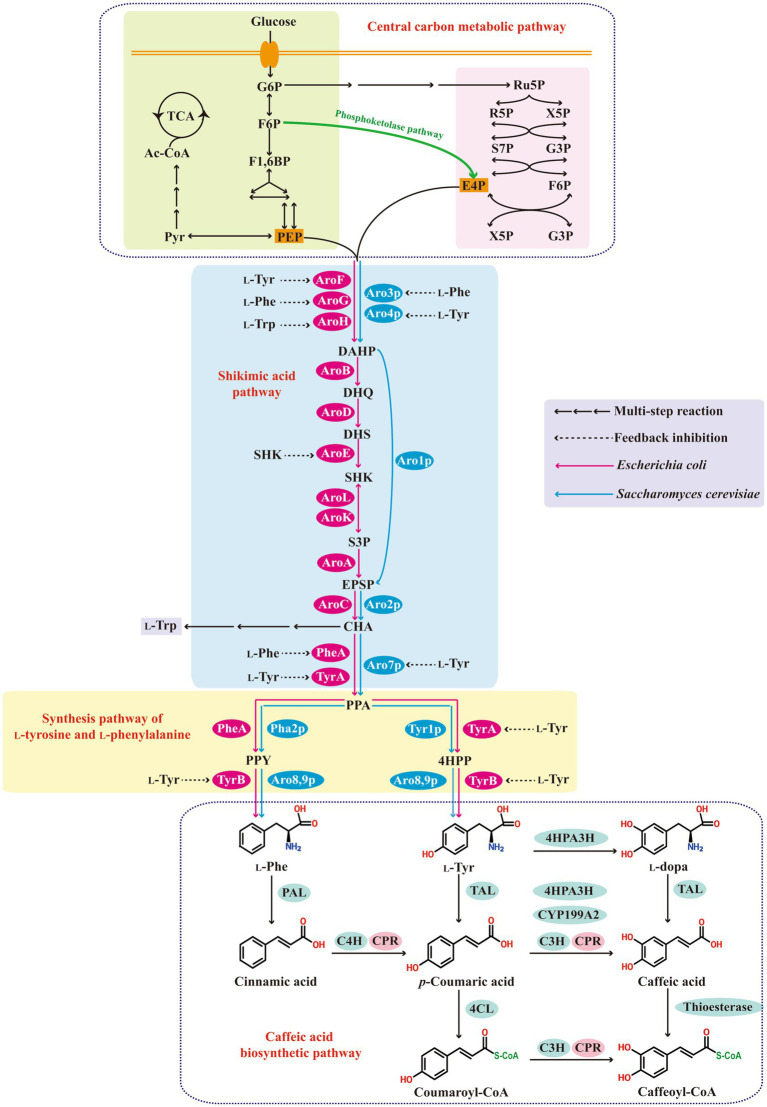
The biosynthetic pathway of caffeic acid and its metabolic regulation. G6P, glucose-6-phosphate; F6P, fructose-6-phosphate; F1,6BP, fructose 1,6-biphosphate; PEP, phosphoenolpyruvate; Pyr, pyruvate; Ac-CoA, acetyl-CoA; TCA tricarboxylic acid cycle; Ru5P, ribulose 5-phosphate; R5P, ribose-5-phosphate; X5P, xylulose-5-phosphate; S7P, sedoheptulose 7-phosphate; G3P, glyceraldehyde 3-phosphate; E4P, erythrose-4-phosphate; DAHP, 3-deoxy-d-arabinoheptulosonic acid-7-phosphate; DHQ, 3-dehydroquinate; DHS, 3-dehydroshikimate; SHIK, shikimate; S3P, shikimate 3-phosphate; EPSP, 5-enolpyruvyl-shikimate 3-phosphate; CHA, chorismic acid; PPA, prephenate; 4HPP, *p*-hydroxyphenylpyruvate; l-Phe, l-phenylalanine; l-Tyr, l-tyrosine; l-Trp, l-tryptophan; PAC, hydroxy-acetaldehyde; AroF, AroG, AroH, Aro3p, and Aro4p, 3-deoxy-d-arabinoheptulosonic acid-7-phosphate synthetase; AroB, 3-dehydroquinate synthetase; AroD, 3-dehydroquinate dehydratase; AroE, shikimate dehydrogenase; AroL and AroK, shikimate kinase; AroA, 5-enolpyruvyl-shikimate 3-phosphate synthetase; Aro1p, pentafunctional enzyme; AroC and Aro2p, chorismic acid synthetase; Aro7p, prephenate mutase; TyrA and Tyr1p, prephenate dehydrogenase; PheA and Pha2p, prephenate dehydratase; TyrB, Aro8p and Aro9p, aromatic aminotransferase; PAL, phenylalanine ammonia-lyase; C4H, cinnamate-4-hydroxylase; TAL, tyrosine ammonia-lyase; C3H, *p*-coumarate-3-hydroxylase; CPR, cytochrome P450 reductase; 4HPA3H, 4-hydroxyphenylacetate 3-hydroxylase; CYP199A2, cytochrome P450 CYP199A2; 4CL, 4-coumarate-CoA ligase.

The biosynthetic pathway of caffeic acid and its metabolic regulation are shown in Figure 1. Glucose is converted into phosphoenolpyruvate and erythrose-4-phosphate via the glycolytic pathway and the pentose phosphate pathway, respectively. Biosynthesis of aromatic amino acids starts with shikimate pathway. The 3-deoxy-d-arabino-heptulosonate-7-phosphate (DAHP) synthase, which is regulated by feedback-inhibition allosteric mechanism, catalyzes the condensation of erythrose-4-phosphate and phosphoenolpyruvate to DAHP. Six enzymatic reactions convert DAHP to chorismite, which is the precursor of aromatic amino acids. These aromatic amino acids are the final products of biosynthetic pathways in most microbial hosts, such as *Escherichia coli* and *S. cerevisiae*. However, in plants and some microorganisms, l-phenylalanine and l-tyrosine are function as intermediates products to synthesize secondary metabolites such as phenylpropanoids. Caffeic acid is mainly produced by the universal route of phenylpropanoid (Lin and Yan, 2012; Rodrigues et al., 2015). First, l-phenylalanine and l-tyrosine are converted to cinnamic acid and *p*-coumaric acid, respectively, by the removal of ammonia under the catalysis of phenylalanine ammonia lyase (PAL) and tyrosine ammonia lyase (TAL). Cinnamic acid is then hydroxylated to *p*-coumaric acid under the action of cinnamate-4-hydroxylase (C4H). Then *p*-coumaric acid is further hydroxylated by coumarate-3-hydroxylase (C3H) to form caffeic acid. Both C4H and C3H are plant-specific cytochrome P450 dependent monooxygenases, and thus, cytochrome P450 reductase (CPR) is required for efficient electron transfer (Kim et al., 2011; Lin and Yan, 2012; Cheniany and Ganjeali, 2016).

## 3 Microbial hosts for caffeic acid biosynthesis

Caffeic acid biosynthesis refers to the implantation of caffeic acid biosynthetic pathway into useful microbial hosts for heterologous expression to produce caffeic acid by metabolic engineering and synthetic biology. The heterologous genes from eukaryotes, prokaryotes, and plants are incorporated into the microbial hosts, which convert cheap renewable carbon sources to caffeic acid through their own metabolic processes. It is very important to select a suitable host for the efficient production of caffeic acid. The commonly used hosts for caffeic acid production are *E. coli* as prokaryotes and *Saccharomyces cerevisiae* as eukaryotes. The main research of caffeic acid biosynthesis in microbial host are shown in Table 1, detailing the microbial hosts utilized, the enzymes or genes introduced, the substrates added to the culture medium, and the yield of caffeic acid.

**Table 1:** Biosynthesis of caffeic acid in engineered microorganisms.

Microbial host	Heterologous enzyme/gene (source)	Host engineering	Substrate	Titer (mg/L)	References
*Escherichia coli* C41 (DE3)	C3H (*Saccharothrix espanaensis*)	—	*p*-Coumaric acid	Not mentioned	Choi et al. (2011)
*E. coli* C41 (DE3)	TAL, C3H (*S. espanaensis*)	Deletion of *tyrR*, and overexpression of *tyrA^fbr^*, *aroG^fbr^*	Glucose	150	Kang et al. (2012)
*E. coli* BL21 (DE3)	CYP199A2 F185, Pux (*Rhodopseudomonas palustris*) Pdr (*Pseudomonas putida*)	—	*p*-Coumaric acid	2,800	Furuya et al. (2012)
*E. coli* BW25113	TAL (*Rhodobacter capsulatus*) 4HPA3H (*E. coli* MG1655)	Overexpression of *tyrA^fbr^*, *aroG^fbr^*, *ppsA*, *tktA*	Glucose + Glycerol	50.2	Lin and Yan (2012)
*E. coli* ATCC31884	TAL (*Rhodotorula glutinis*) 4HPA3H (*E. coli* MG1655)	Deletion of *pheLA*, disruption of *tyrA*, and overexpression of *tyrA^fbr^*, *aroG^fbr^*, *ppsA*, *tktA*	Glucose + Glycerol	766.68	Huang et al. (2013)
*E. coli* BW25113	4HPA3H (*E. coli* MG1655)	—	*p*-Coumaric acid	3,820	
*E. coli* rpoA14(DE3)	TAL (*R. glutinis*) C3H (*S. espanaensis*)	—	Glucose	106	Zhang and Stephanopoulos (2013)
			Xylose	70	
*E. coli* BL21 Star (DE3)	4HPA3H (*Pseudomonas aeruginosa* PAO1)	—	*p*-Coumaric acid	10,200	Furuya and Kino (2014)
*E. coli* K-12 MG1655 (DE3)	TAL (*R. glutinis*) C3H (*S. espanaensis*)	—	l-Tyrosine	180	Rodrigues et al. (2015)
	TAL (*R. glutinis*) CYP199A2 F185, Pux (*R. palustris*) Pdr (*P. putida*)			280	
*E. coli* AN219	TAL (*Streptomyces* sp. WK-5344) 4HPA3H (*P. aeruginosa* PAO1)	—	Cellulose from kraft pulp	233	Kawaguchi et al. (2017)
*E. coli* K-12 MG1655(DE3)	TAL (*Flavobacterium johnsoniae*) CYP199A2 F185L NΔ7, Pux and Pdr (*R. palustris*)	—	Glucose	47	Haslinger and Prather (2020)
*E. coli* ATCC31882	TAL (*R. glutinis*) C3H (*S. espanaensis*) HpaC (*P. aeruginosa*)	Deletion of *trpE*, disruption of *pheA*, and overexpression of *aroF*, *aroG*, *tyrR*, *tyrA*, *trpE*, *tyrA^fbr^*	Glucose	6,170	Sakae et al. (2023)
*Saccharomyces cerevisiae* BY4741	TAL (*Rhodosporidium toruloides*) HpaB (*P. aeruginosa*) HpaC (*Salmonella enterica*)	—	l-Tyrosine	289.4	Liu L. Q. et al. (2019)
*S. cerevisiae* BY4742	TAL (*Rhodobacter capsulatus*) C3H, CPR1 (*Arabidopsis thaliana*)	Deletion of *ARO10*, PDC5, and overexpression of *ARO4^fbr^*, *ARO7^fbr^*	Glucose	11.432	Li et al. (2020)
*S. cerevisiae* BY4741	TyrC (*Zymomonas mobilis*) TAL (*R. glutinis*) HpaB (*P. aeruginosa*) HpaC (*S. enterica*)	Deletion of *ARO3*, *ARO10*, *ARO80*, and overexpression of *ARO4^fbr^*, *ARO7^fbr^*	Glucose	569.0	Zhou et al. (2021)
*S. cerevisiae* NKC6	TAL (*Rhodobacter capsulatus*) C3H and CPR1 (*A. thaliana*)	Deletion of *TPI1*	Glucose	15.1	Qi et al. (2022)
*S. cerevisiae* NK-B2 and SK10-3	TAL (*Rhodobacter capsulatus*) HpaB (*P. aeruginosa*) HpaC (*S. enterica*)	—	Carboxymethyl-cellulose	16.91	Cai et al. (2022)
CEN.PK113-11C*	AroL (*E. coli*) TAL (*F. johnsoniae*) PAL1 (*Sorghum bicolor*) CPR1 (*A. thaliana*) C4H1, C4H2, C3H3 (*Populus trichocarpa*) HpaB (*P. aeruginosa*) HpaC (*S. enterica*) PDH1 (*Medicago truncatula*) PTA (*Clostridium kluyveri*) XFPK (*Leuconostoc mesenteroides*) FADS (*Thermotoga maritima*) RIBBA (*Bacillus subtilis*)	Deletion of *ARO10*, *PDC5*, and *GPP1*, and overexpression of *ARO4^fbr^*, *ARO7^fbr^*, *ARO1*, *ARO2*, *ARO3*, *PHA2*, *TAL1*, *TKL1*	Glucose	5,500	Chen et al. (2022b)
*Streptomyces fradiae* XKS	TAL, C3H (*S. espanaensis*)	—	l-Tyrosine	Not mentioned	Berner et al. (2006)
*Synechocystis* sp. PCC 6803	C3H (*A. thaliana*)	—	*p*-Coumaric acid	7.2	Xue et al. (2014)

### 3.1 *E. coli* host

*Escherichia coli* is taken as an ideal host for the engineering and production of different biomolecules due to its rapid growth rate, short life cycle, low cultivation cost, clear genetic background, easy genetic manipulation, and good ability for enzyme overexpression (Makrides, 1996; Wu et al., 2013; Liu et al., 2016; Wang et al., 2016). Moreover, l-tyrosine and *p*-coumaric acid, the key precursors of caffeic acid, are crucial for increasing caffeic acid production and easy to be improved in *E. coli* (Shrestha et al., 2019). In addition, *E. coli* is better suited for caffeic acid production than yeast due to its superior tolerance to *p*-coumaric acid (Shin et al., 2011; Zhang and Stephanopoulos, 2013; Li et al., 2020). The first attempt to produce caffeic acid in *E. coli* was described by Choi et al. (2011), who introduced C3H from *Saccharothrix espanaensis* into *E. coli* C41 (DE3) strain and achieved caffeic acid with the supplementation of precursor *p*-coumaric acid. However, a significant limitation of using *E. coli* as an expression host is its lack of capacity for post-translational modifications, which are typically necessary for the correct folding and functional activity of recombinant proteins.

### 3.2 *Saccharomyces cerevisiae* host

Like *E. coli*, *S. cerevisiae* is also taken as an ideal host for the engineering and production of different biomolecules due to its easy growth, low cultivation cost, clear genetic background, and easy genetic manipulation (Yesilirmak and Sayers, 2009; Borodina and Nielsen, 2014). Furthermore, *S. cerevisiae* is a generally recognized as safe (GRAS) host and widely used in food markets and pharmaceutical products (Krivoruchko and Nielsen, 2015; Pereira et al., 2019). One major advantage of using *S. cerevisiae* rather than *E. coli* as a host is that its subcellular compartmentation is similar to that of plant cells, which make it has ability to perform post-translational modifications (Rainha et al., 2020). Thus *S. cerevisiae* can adequately express membrane proteins such as cytochrome P450 (Kim et al., 2011; Koopman et al., 2012; Sadeghi et al., 2013; Bostick et al., 2016). Li et al. (2020) determined that the codon optimized C3H gene from *Arabidopsis thaliana* can successfully express and hydroxylate the substrate *p*-coumaric acid to caffeic acid in *S. cerevisiae* strain. Nevertheless, *S. cerevisiae* is more sensitive to high concentration of *p*-coumaric acid (Shrestha et al., 2019).

### 3.3 Other hosts

Apart from *E. coli* and *S. cerevisiae*, *S. fradiae* XKS and cyanobacterium *Synechocystis* PCC 6803 have also been engineered to produce caffeic acid. Berner et al. (2006) have successfully expressed TAL and C3H from *S. espanaensis* in *S. fradiae* XKS, and achieved caffeic acid with the supplementation of l-tyrosine. Xue et al. (2014) introduced C3H from *A. thaliana* into cyanobacterium *Synechocystis* PCC 6803 to obtain 7.2 mg/L caffeic acid, when supplemented with *p*-coumaric acid.

## 4 Metabolic engineering to enhance caffeic acid production

Metabolic engineering of caffeic acid production in microbial hosts has achieved great progress in recent years. The main strategies for increasing caffeic acid productivity using microbial cell factories included selecting and optimizing heterologous enzymes via enzyme engineering, increasing the metabolic flux to caffeic acid via pathway engineering, enhancing the supply and recycling of cofactor via cofactor engineering, and optimizing the production process via fermentation engineering.

### 4.1 Enzyme selection and engineering

The heterologous enzymes needed for the synthesis of caffeic acid in microorganisms mainly include ammonia-lyase and hydroxylase.

#### 4.1.1 Ammonia-lyase

TAL catalyzes the deamination of l-tyrosine to produce *p*-coumaric acid. PAL catalyzes the deamination of l-phenylalanine to produce cinnamic acid, which is then hydroxylated at the 4-position of the benzene ring to form *p*-coumaric acid by the action of C4H and its associated CPR. Jendresen et al. (2015) screened and expressed 22 wild type or artificially modified TAL and PAL genes for *p*-coumaric acid production, and found that enzymes from *Flavobacterium johnsoniae* and *Herpetosiphon aurantiacus* resulted in high *p*-coumaric acid production in *E. coli*, *S. cerevisiae*, and *Lactococcus lactis* strains. TAL from *S. espanaensis* can also result in high production of *p*-coumaric acid in *E. coli* strain. Kawaguchi et al. (2017) found that TAL (*fevV*) from *Streptomyces* sp. WK-5344 can be successfully expressed and convert l-tyrosine to *p*-coumaric acid in *E. coli* strain.

#### 4.1.2 Hydroxylase

C3H catalyzes the hydroxylation of *p*-coumaric acid at 3-position of the benzene ring to form caffeic acid. The hydroxylase which can function in *E. coli* mainly contains *S. espanaensis* C3H, *Rhodopseudomonas palustris* cytochrome P450 CYP199A2, and *E. coli* endogenous 4-hydroxyphenylacetate 3-hydroxylase (4HPA3H). Choi et al. (2011) used an *E. coli* strain C41 (DE3) harboring C3H from *S. espanaensis* and achieved caffeic acid in the presence of *p*-coumaric acid. Furuya et al. (2012) achieved the caffeic acid with an *E. coli* strain BL21 (DE3) harboring cytochrome P450 CYP199A2 from *R. palustris* and its associated putidaredoxin reductase gene (*pdR*) from *Pseudomonas putida* and palustrisredoxin gene (*pux*) from *R. palustris* with the supplementation of *p*-coumaric acid. To enhance the activities of CYP199A2, the F185 residue of CYP199A2 was substituted with Tyr (F185Y), Trp (F185W), Val (F185V), Leu (F185L), Ile (F185I), Gly (F185G), Ala (F185A), Ser (F185S), or Thr (F185T), respectively. The F185L mutant was confirm to have the highest hydroxylation activity for *p*-coumaric acid, which was 5.5 times higher than that of wild-type CYP199A2, and the production of caffeic acid from *p*-coumaric acid using the F185L whole-cell catalyst reached 2.8 g/L (Furuya et al., 2012). Further, Haslinger and Prather (2020) enhanced the efficiency of the electron transfer step from the two redox partner proteins to CYP199A2 F185L through using the natural redox system, tethering the redox complex by creating genetic fusions with high-affinity tethering domains, and supplying extra gene copies coding for *pux*. About 47 mg/L caffeic acid was obtained from the *E. coli* MG1655 (DE3) strain incorporating TAL from *F. johnsoniae*, CYP199A2 F185L, tethered redox complex, and extra gene copies coding for *pux* with glucose as the only carbon source (Haslinger and Prather, 2020). It has been demonstrated that CYP199A2 F185L with its redox partners allowed a higher caffeic acid titer when compared with C3H from *S. espanaensis* (Rodrigues et al., 2015). 4HPA3H complex, which is composed of 4-hydroxyphenylacetate 3-monooxygenase (HpaB) and NADPH-flavin oxidoreductase (HpaC), can hydroxylate a broad range of substrates at the 3-position, including l-tyrosine, tyrosol, and *p*-hydroxyphenylacetate (Galan et al., 2000). Considering the structural similarity of *p*-coumaric acid to l-tyrosine, tyrosol, and *p*-hydroxyphenylacetate, researchers hypothesized that 4HPA3H should be able to catalyze *p*-coumaric acid as well (Furuya and Kino, 2014). Lin and Yan (2012) first identified that the *E. coli* native 4HPA3H could convert *p*-coumaric acid to caffeic acid efficiently. Huang et al. (2013) overexpressed *HpaBC* with a high-copy plasmid pZE12-luc in *E. coli* BW25113 strain, and produced 3.82 g/L caffeic acid from 4 g/L *p*-coumaric acid in shake flasks after 24 h cultivation.

The hydroxylase which can function in *S. cerevisiae* mainly contains 4HPA3H derived from different species and C3H from *A. thaliana*. Li et al. (2020) overexpressed *A. thaliana*-derived C3H and CPR1 with a high-copy plasmid pLC41 in yeast BY4742, and produced 18.1 mg/L caffeic acid from 600 mg/L *p*-coumaric acid in shake flasks after 120 h cultivation, indicating the *A. thaliana*-derived C3H can be expressed in *S. cerevisiae* strain and catalyze the conversion of *p*-coumaric acid to caffeic acid. Liu L. Q. et al. (2019) constructed functional 4HPA3H in *S. cerevisiae* strain by recruiting HpaB and HpaC from several bacteria, and obtained the highest production of caffeic acid (289.4 mg/L) from 500 mg/L l-tyrosine by co-overexpressing of TAL from *Rhodosporidium toruloides* and the enzyme combination of HpaB from *Pseudomonas aeruginosa* and HpaC from *Salmonella enterica* in shake flasks after 96 h cultivation.

### 4.2 Pathway engineering

There is an endogenous aromatic amino acid biosynthesis pathway in microbial hosts, such as *E. coli* and *S. cerevisiae* (Figure 1). However, this pathway is mostly regulated by feedback inhibition of allosterically controlled key enzymes and the accumulation of aromatic amino acid is very low, thus the enhancement of metabolic flux to l-phenylalanine and l-tyrosine is vitally important for caffeic acid production. Meanwhile, microbial hosts, such as *E. coli* and *S. cerevisiae*, lack a phenylpropanoid metabolism pathway, thus the construction of heterologous synthesis pathway of caffeic acid is essential for bioconversion or *de novo* biosynthesis of caffeic acid. The two main goals within this step include the enhancement of metabolic flux to l-phenylalanine and l-tyrosine and the functional introduction of heterologous synthesis pathway of caffeic acid.

#### 4.2.1 The enhancement of metabolic flux to l-phenylalanine and l-tyrosine

The mainly strategies to enhance the endogenous metabolic flux to l-phenylalanine and l-tyrosine biosynthesis contain the enhancement of precursor supply, elimination of enzyme feedback inhibition regulation, blocking of competitive pathways, and balance of the ratio between phosphoenolpyruvate and erythrose 4-phosphate.

In *E. coli* strain, Kang et al. (2012) constructed a l-tyrosine high-producing strain by knocking out *tyrR* to remove the repression of TyrR protein to l-tyrosine metabolic pathway, overexpressing *aroG^fbr^* to relieve the feedback inhibition of l-phenylalanine on AroG protein, and overexpressing *tyrA^fbr^* mutant to relieve the feedback inhibition of l-tyrosine on TyrA protein, and achieved a 2.6-fold higher caffeic acid production (150 mg/L) from glucose by overexpressing TAL and C3H from *S. espanaensis* in this l-tyrosine high-producing strain. Furthermore, the production of l-tyrosine was increased by overexpressing *ppsA* and *tktA* to enhance the supply of precursors phosphoenolpyruvate and erythrose 4-phosphate, disrupting *pheA* to block the competitive pathway of l-phenylalanine, and disrupting *tyrA* to eliminate feedback inhibition of l-tyrosine (Lin and Yan, 2012; Huang et al., 2013).

In *S. cerevisiae* strain, Li et al. (2020) constructed a l-tyrosine high-producing strain by overexpressing the mutated feedback-resistant *ARO4^K229L^* and *ARO7^G141S^* to alleviate feedback inhibition by l-tyrosine on DAHP synthase and chorismite mutase, and knocking out *ARO10* and *PDC5* to avoid production of aromatic alcohols and direct the pathway flux to l-tyrosine, and resulted in a 14.1-fold higher caffeic acid production from glucose by overexpressing TAL from *Rhodobacter capsulatus* and C3H from *A. thaliana* in this l-tyrosine high-producing strain. The activity of endogenous prephenate dehydrogenase Tyr1p in *S. cerevisiae* is feedback-inhibited by l-phenylalanine, while the cyclohexadienyl dehydrogenase TyrC from *Zymomonas mobilis* can also catalyze the conversion of prephenic acid to 4-hydroxyphenylpyruvate without being feedback-inhibited by l-phenylalanine (Gold et al., 2015; Mao et al., 2017). Zhou et al. (2021) engineered a high-throughput caffeic acid synthetic pathway by knocking out *ARO3* and *ARO10*, and inducibly overexpressing *ARO4^K229L^*, *ARO7^G141S^*, TyrC, TAL from *Rhodotorula glutinis*, hpaB from *P. aeruginosa*, and hpaC from *S. enterica*, achieving 569.0 mg/L of caffeic acid from glucose in shake flask fermentation, with a 1.6-fold increase in production due to the enhanced l-tyrosine metabolic flux. Liu Q. L. et al. (2019) engineered the aromatic amino acid biosynthesis pathway by introducing a phosphoketolase-based pathway to divert glycolytic flux toward erythrose 4-phosphate formation, and optimizing the carbon distribution between aromatic amino acid biosynthesis pathway and glycolysis pathway through replacing the promoters of several important genes at key nodes between these two pathways. Furthermore, they simultaneously introduced a single heterologous enzymatic step from l-tyrosine (TAL from *F. johnsoniae*) and two enzymatic steps from l-phenylalanine (PAL2, C4H and CPR2 from *A. thaliana*, a cytochrome B5 from *S. cerevisiae*) in this strain to produce a maximum *p*-coumaric acid titer of 12.5 g/L, providing a new strategy for constructing platform strain for high l-phenylalanine and l-tyrosine yields (Liu Q. L. et al., 2019).

#### 4.2.2 The introduction of heterologous synthesis pathway of caffeic acid

In prokaryotic microorganisms, caffeic acid is commonly synthesized by a l-tyrosine derived pathway due to the difficult expression of plant-specific cytochrome P450 enzymes in prokaryotic microbial system, whereas in eukaryotic microorganisms, caffeic acid can be synthesized by a l-tyrosine derived pathway or a l-phenylalanine derived pathway. l-Phenylalanine or l-tyrosine are first converted to *p*-coumaric acid. Then, *p*-coumaric acid is further converted to caffeic acid by sam5-encoded C3H from the *S. espanaensis*, or cytochrome P450 CYP199A2 from *R. palustris*, or 4HPA3H from different microbial sources (Furuya et al., 2012; Lin and Yan, 2012; Li et al., 2020). The l-phenylalanine derived pathway was far more efficient compared with the l-tyrosine derived pathway. Liu Q. L. et al. (2019) introduced a l-phenylalanine derived pathway containing PAL2, C4H, and CPR2 from *A. thaliana* and cytochrome B5 (CYB5) from *S. cerevisiae*, as well as a l-tyrosine derived pathway containing highly specific TAL from *F. johnsoniae* in *S. cerevisiae* strain, respectively, and found that the l-phenylalanine derived pathway can produced 337.6 mg/L *p*-coumaric acid under glucose-limited conditions, which was 26.17-fold higher than that of the l-tyrosine derived pathway. Chen et al. (2022b) introduced a l-phenylalanine derived pathway containing *Sorghum bicolor* PAL1, *A. thaliana* CPR1, and an identified Cyp complex (containing C4H2, C4H1, and C3H3 from *Populus trichocarpa*) into *S. cerevisiae* strain expressing TAL from *F. johnsoniae*, and resulted in a 5.66-fold increase in *p*-coumaric acid accumulation in shake-flask cultivation. In addition, l-tyrosine can also be first converted to l-dopa by 4HPA3H, and then TAL converts l-dopa to caffeic acid. A potential caffeic acid biosynthesis pathway is to convert l-tyrosine to *p*-coumaric acid, then to coumaroyl-CoA, to caffeoyl-CoA, and finally to caffeic acid by TAL, 4-coumarate-CoA ligase, C3H, and thioesterase, respectively (Zhang and Stephanopoulos, 2013) (Figure 1).

### 4.3 Cofactor engineering

At present, researchers mainly improve the microbial synthesis of caffeic acid products by selecting and optimizing heterologous enzymes of caffeic acid synthesis pathway and enhancing the metabolic flux of l-tyrosine and l-phenylalanine in host cells, while neglecting the role of cofactors in the biosynthesis of natural products. In the biosynthetic pathway of caffeic acid, HpaBC is a two-component monooxygenase. HpaB performs the hydroxylation reaction with the aid of FADH_2_ cofactor, whereas HpaC, serving as a flavin reductase, is responsible for the conversion of FAD to FADH_2_ through the consumption of NADH (Galan et al., 2000; Xun and Sandvik, 2000; Phillips and Shephard, 2008). This interplay ensures the seamless progression of the monooxygenase reaction. Meanwhile, the regeneration of NADPH is crucial for the function of cytochrome P450 enzymes in the synthesis of the precursor *p*-coumaric acid (Yang et al., 2020). Chen et al. (2022a) systematically optimized the supply and recycling pathways of cofactors NADPH and FADH_2_ in the *S. cerevisiae* strain, increasing the titer of caffeic acid by 31% to 5.5 g/L, which is the highest titer reported for caffeic acid. Fre, a flavin reductase enzyme in *E. coli*, catalyzes the reduction of flavin to FADH_2_ by utilizing NAD (P) H as the electron donor (Fontecave et al., 1987; Fieschi et al., 1995). Zhou et al. (2022) increased the hydroxylation efficiency of HpaBC by 810% in *E. coli* strain through the fusion expression of Fre and HpaBC, which accelerate the regeneration of FADH_2_. Effendi and Ng (2023) enriched FADH_2_ cofactor pools via deploying *fre* gene in *E. coli* strain, which improve ferulic acid titer by 37.14%.

### 4.4 Fermentation engineering

#### 4.4.1 Microbial co-culture strategy

Microorganism co-cultivation can not only fully utilize the advantages of various microorganisms but also alleviate the metabolic stress on single microbial cells, and is being increasingly applied to the biosynthesis of natural products (Nakayama et al., 2011; Goers et al., 2014; Zhang et al., 2017; Rodrigues et al., 2020). Cai et al. (2022) co-cultured engineered *S. cerevisiae* strain SK10-3 harboring the carboxymethyl cellulose hydrolysis pathway composed of *Talaromyces emersonii CBHI*, *Trichoderma reesei EGII*, and *Aspergillus aculeatus BGLI* with engineered *S. cerevisiae* strain NK-B2b harboring *p*-coumaric acid synthesis pathway composed of *R. capsulatus TAL* and produced 71.71 mg/L *p*-coumaric acid from carboxymethyl cellulose, which is 155.9-fold higher than the mono-culture of SK10-3b harboring both carboxymethyl cellulose hydrolysis pathway and *p*-coumaric acid synthesis pathway. Then, the *de novo* biosynthesis of caffeic acid from carboxymethyl-cellulose was achieved through the co-culture of engineered *S. cerevisiae* strains SK10-3, NK-B2b, and NK-B2d harboring caffeic acid synthesis pathway composed of *P. aeruginosa hpaB* and *S. enterica hpaC*. This strategy provides a novel approach for the low-cost and high-efficiency synthesis of caffeic acid (Cai et al., 2022).

#### 4.4.2 Optimization of the fermentation medium

Research has found that the composition of culture medium, especially the carbon source, greatly affect the yield of caffeic acid. Furuya et al. (2012) confirmed that glycerol was more effective than glucose in supporting the hydroxylation carried out by *E. coli* cells expressing the F185L mutant of CYP199A2. By using the F185L whole-cell catalyst in the presence of glycerol, the production of caffeic acid from *p*-coumaric acid reached 2.8 g/L, 1.65-fold higher than in the presence of glucose (Furuya et al., 2012). Zhang and Stephanopoulos (2013) evaluated the synthesis of caffeic acid by recombinant *E. coli* strain under various conditions and discovered that glucose was more suitable as a carbon source than xylose. Furthermore, when comparing the MOPS medium, rich medium, and synthetic medium, it was found that the rich medium yielded the highest caffeic acid titer, whereas the synthetic medium resulted in the highest specific titer (the ratio of caffeic acid production to cell density) (Zhang and Stephanopoulos, 2013). Li et al. (2020) found that engineered strains of *S. cerevisiae* exhibited greater growth density and 4.1-fold higher caffeic acid production in the rich medium YPD containing 4% glucose compared to the synthetic medium SC-Ura-His containing 2% glucose. Kawaguchi et al. (2017) produce 233 mg/L of caffeic acid from inedible cellulose through simultaneous saccharification and fermentation (SSF) reactions using a recombinant *E. coli* strain. Sakae et al. (2023) produce 6.17 g/L of caffeic acid using a modified M9Y medium with 50 g/L glucose in a jar fermenter.

## 5 Conclusion and future prospect

Caffeic acid, an important natural compound of plants, is not only widely used in the pharmaceutical and cosmetic industries but also frequently serves as an intermediate in the biosynthesis of many high-value natural compounds, such as caffeic acid phenethyl ester and chlorogenic acid, thus possessing significant commercial value. In recent years, the demand for caffeic acid has been growing continuously with the improvement of people’s living standards. The construction of cell factories for the synthesis of caffeic acid provides a green and sustainable alternative to traditional plant extraction and chemical synthesis methods. By utilizing renewable resources, avoiding the use of toxic solvents and complex chemical reactions, and minimizing waste generation, the microbial production of caffeic acid not only reduces environmental pollution but also provides significant support for the development of a circular bioeconomy. In recent years, microbial biosynthesis of caffeic acid has achieved remarkable success, with the highest reported *de novo* production titer reaching 6.17 g/L (Sakae et al., 2023). Microbial synthesis of caffeic acid is expected to make further progress in the following areas.

### 5.1 Enhancement of metabolic flux to caffeic acid biosynthesis pathway

The low activity of heterologous enzymes in microbial hosts and the insufficient supply of precursor molecules by microbial metabolism are the main factors limiting the concentration of caffeic acid. Therefore, it is necessary to combine the regulation of aromatic amino acid metabolic pathways, the screening of high-activity caffeic acid synthetase, and the enhancement of cofactors supply and recycling together to strengthen the metabolic flux of caffeic acid biosynthesis in microbial cells. In recent years, the application of machine learning algorithms in the learning phase of the design-build-test-learn cycle has been increasing. Through machine learning, it is possible to design and optimize biosynthetic pathways, analyze the relationship between protein structure and function, and optimize protein design (Kim et al., 2020; Cuperlovic-Culf et al., 2023; Sieow et al., 2023). To improve the activity of enzymes, protein engineering, particularly directed evolution, can be employed. Luo et al. (2021) reported a deep-learning algorithm named evolutionary context-integrated neural network, which leverages evolutionary contexts to predict functional fitness for protein engineering, enabling accurate mapping from sequence to function and generalization from low-order to higher-order mutants. The optimization of each step in the biosynthetic pathway should be carried out to ensure that every metabolite and precursor is directed toward the final product. Additionally, a comprehensive understanding of the synthetic and molecular biology of each component involved in caffeic acid biosynthesis, including the genome, transcriptome, proteome, and metabolome, can significantly enhance the efficiency of production from microbial platforms.

### 5.2 Screening and construction of suitable microbial hosts

Currently, the common microbial host for caffeic acid biosynthesis are *E. coli* and *S. cerevisiae*. On the one hand, C4H, which is required for the synthesis of caffeic acid, belongs to the cytochrome P450 dependent monooxygenases and is difficult to express in *E. coli*. Therefore, caffeic acid can only be synthesized through the l-tyrosine derived pathway in *E. coli*. As a eukaryote, *S. cerevisiae* has certain advantages in expressing plant-derived P450 enzymes. In *S. cerevisiae*, caffeic acid can be synthesized through both the l-tyrosine derived pathway and the l-phenylalanine derived pathway, which greatly increases the yield of caffeic acid. On the other hand, high concentrations of caffeic acid and *p*-coumaric acid have certain inhibitory effects on the growth of *E. coli* and *S. cerevisiae*. With the continuous improvement of caffeic acid production, the tolerance of microbial host to caffeic acid and *p*-coumaric acid will become a restrictive factor. Research indicates that *E. coli* has a higher tolerance to caffeic acid and *p*-coumaric acid than *S. cerevisiae*. In the future, the mechanisms by which caffeic acid and *p*-coumaric acid inhibit the growth of microbial hosts can be deeply analyzed, and the microbial hosts can be modified in a “top-down” manner or engineered through “bottom-up” synthetic biology design to construct strains with high tolerance to caffeic acid and *p*-coumaric acid. Some approaches, such as adaptive laboratory evolution and multi-functional genome-wide CRISPR system, can be designed to enhance the tolerance of microbial host to caffeic acid and *p*-coumaric acid while enhancing the yield of caffeic acid. Wang et al. (2021) employed an adaptive laboratory evolution approach with increasing concentrations of ferulic acid to significantly enhance the tolerance of the *Yarrowia lipolytica* strain XYL+ to ferulic acid and identified the tolerance-related genes via transcriptomic analysis of the evolved strain. Lian et al. (2019) enhanced the furfural tolerance of yeast using the multi-functional genome-wide CRISPR system, which can identify genetic determinants of complex phenotypes that have not been previously characterized, especially those genes that interact synergistically when perturbed to different expression levels. In addition, a co-culture system of strains can also be established to fully leverage the advantages of different microbial hosts for the production of caffeic acid.

### 5.3 Establishment of a low cost, high efficiency industrial production process of caffeic acid

At present, the biosynthesis of caffeic acid is still at the laboratory level. The carbon source for microbial fermentation is mostly glucose. Compared to glucose, cellulose is mainly sourced from low-cost agricultural residues, such as straw and wood chips. With the advancement of cellulase technology and the optimization of pretreatment processes, utilizing cellulose as a carbon source for the industrial-scale production of bio-products may not only reduce production costs but also provide support for the development of a circular economy. In the future, it is possible to develop cheap fermentation raw materials such as cellulose, optimize and scale up the fermentation process, reduce the difficulty and cost of separation and purification, and establish an industrial production process for caffeic acid that is low-cost and highly efficient.
